# Relationship Between Body Mass Index and Chest Compression Quality Following Standardized Basic Life Support Training in Nurses: A Prospective Simulation Study

**DOI:** 10.3390/healthcare14162562

**Published:** 2026-08-16

**Authors:** Tuba Kuvvet Yoldaş, Gözde Gürsoy Çirkinoğlu, Canan Salman Önemli

**Affiliations:** Department of Anesthesiology and Reanimation, Izmir City Hospital, 35540 Izmir, Türkiye; dr.gozde.gursoy@gmail.com (G.G.Ç.); canan_ege_35@hotmail.com (C.S.Ö.)

**Keywords:** body mass index, basic life support, cardiopulmonary resuscitation, chest compression, nurses, simulation

## Abstract

**Background:** High-quality chest compressions are considered a fundamental component of effective cardiopulmonary resuscitation (CPR). Although body mass index (BMI) has been suggested to be associated with chest compression performance, the available evidence remains unclear, particularly among healthcare professionals evaluated under standardized training conditions. This study aimed to investigate the association between BMI and chest compression quality following standardized Basic Life Support (BLS) training in nurses. **Methods:** This prospective simulation study included 284 nurses who completed a standardized 45 min BLS training program. Chest compression performance was assessed individually using an AmbuMan^®^ Basic CPR manikin during two minutes of uninterrupted chest compressions. Adequate compression rate, adequate compression depth, and complete chest recoil were evaluated according to the manufacturer’s performance criteria based on current ERC and AHA guidelines. Participants were classified into three BMI groups: <18.5, 18.5–24.9, and ≥25 kg/m^2^. Multivariable logistic regression was performed to evaluate the independent association between BMI and each chest compression quality parameter, adjusting for age, sex, nursing experience, previous BLS training, and working in an intensive care unit. **Results:** Participants with a BMI < 18.5 kg/m^2^ had significantly lower rates of adequate compression rate (15.0%) and adequate compression depth (45.0%) than those in the other two BMI groups (both *p* < 0.001). Complete chest recoil also differed significantly among BMI groups (*p* = 0.004). After adjustment for age, sex, nursing experience, previous BLS training, and working in an intensive care unit, BMI remained independently associated with adequate compression rate (adjusted OR 1.32, 95% CI 1.18–1.49; *p* < 0.001) and adequate compression depth (adjusted OR 1.29, 95% CI 1.14–1.47; *p* < 0.001), but not with complete chest recoil (adjusted OR 1.12, 95% CI 0.99–1.26; *p* = 0.072). **Conclusions:** Following standardized BLS training, nurses with a BMI < 18.5 kg/m^2^ showed lower rates of adequate compression rate and depth than those with higher BMI values, while BMI was not independently associated with complete chest recoil. These findings suggest an association between lower BMI and poorer performance in selected chest compression parameters within this simulation setting.

## 1. Introduction

Cardiac arrest remains one of the leading causes of mortality worldwide, and early, effective intervention is a major determinant of patient survival. Cardiopulmonary resuscitation (CPR) is a fundamental life-saving intervention for patients experiencing cardiac arrest. Numerous studies have demonstrated that high-quality chest compressions are closely associated with return of spontaneous circulation, survival to hospital discharge, and favorable neurological outcomes [1,2].

The effectiveness of CPR depends largely on the quality of chest compressions. Adequate compression depth, an appropriate compression rate, and complete chest recoil after each compression are considered essential components of effective resuscitation. Current guidelines from the American Heart Association (AHA) and the European Resuscitation Council (ERC) recommend delivering uninterrupted, high-quality chest compressions at the recommended depth and rate to maintain adequate coronary and cerebral perfusion during resuscitation [3,4].

In addition to theoretical knowledge and technical proficiency, CPR performance may also be influenced by the physical characteristics of the rescuer. Anthropometric factors such as height, body weight, muscle strength, physical fitness, and body mass index (BMI) have been suggested to affect the mechanical force generated during chest compressions and, consequently, the quality of CPR. However, the relationship between BMI and chest compression quality remains controversial. Previous studies have reported that rescuers with a higher BMI may achieve the recommended compression depth more easily by utilizing greater body weight during compressions [5,6,7]. Conversely, this potential advantage may be accompanied by more rapid fatigue, reduced consistency of compression rate, or impaired complete chest recoil due to increased residual force exerted on the patient’s chest [5,8]. A recent systematic review further demonstrated that several physical characteristics, including BMI, body weight, muscle strength, physical activity, and body composition, are associated with CPR quality. However, it also highlighted considerable heterogeneity among previous studies regarding the physical characteristics evaluated, CPR quality outcomes, and assessment protocols, resulting in inconsistent evidence and emphasizing the need for further research in this field [9].

Nurses play a pivotal role in the management of in-hospital cardiac arrest by initiating basic life support and maintaining high-quality chest compressions until advanced resuscitation measures are established. Therefore, identifying factors that influence CPR performance among nurses is important for optimizing training strategies and improving the overall quality of resuscitation [10,11]. Because CPR performance is influenced not only by physical characteristics but also by knowledge and technical skills, all participants in the present study completed the same standardized Basic Life Support (BLS) training before the simulation assessment. This approach was intended to reduce variability related to CPR training, thereby enabling evaluation of the association between BMI and chest compression quality under standardized educational conditions.

The aim of this prospective simulation study was to investigate the association between body mass index (BMI) and the three core components of chest compression quality—compression rate, compression depth, and complete chest recoil—among nurses following standardized Basic Life Support (BLS) training. We hypothesized that body mass index would be associated with chest compression quality following standardized Basic Life Support training in nurses.

## 2. Materials and Methods

### 2.1. Study Design and Participants

This prospective simulation study was conducted between May and June 2026 to investigate the association between body mass index (BMI) and chest compression quality parameters among nurses following standardized Basic Life Support (BLS) training. The study population consisted of nurses who attended the annual in-service BLS training program organized at our tertiary care hospital. Ethical approval was obtained from the Izmir City Hospital Clinical Research Ethics Committee (Approval No. 2026/295), and written informed consent was obtained from all participants before enrollment. Eligible participants were actively employed nurses who attended the BLS training course and voluntarily agreed to participate in the study. Individuals with chronic musculoskeletal or neurological disorders that could interfere with chest compression performance, as well as those who declined participation, were excluded. Eligible nurses attending the scheduled annual in-service Basic Life Support training sessions during the study period were consecutively invited to participate in the study.

### 2.2. Demographic and Anthropometric Data

Baseline demographic characteristics, including age, sex, height, body weight, and duration of professional nursing experience, were recorded. Body mass index was calculated by dividing body weight in kilograms by the square of height in meters (kg/m^2^). Participants were classified according to the World Health Organization (WHO) BMI thresholds into three categories: <18.5 kg/m^2^, 18.5–24.9 kg/m^2^, and ≥25 kg/m^2^. Because only 13 participants met the criterion for obesity (BMI ≥30 kg/m^2^), maintaining obesity as a separate category would have resulted in a small subgroup and limited the reliability of between-group statistical comparisons. Therefore, participants with overweight and obesity were combined into a single BMI ≥ 25 kg/m^2^ category for statistical analysis.

### 2.3. Basic Life Support Training

All participants received a standardized 45 min Basic Life Support training session based on the current recommendations of the European Resuscitation Council (ERC) and the American Heart Association (AHA). The training consisted of a theoretical lecture followed by a practical demonstration using a CPR training manikin. Immediately after completing the training program, each participant underwent an individual assessment of chest compression performance. The standardized BLS training sessions were delivered by two board-certified anesthesiologists who were certified BLS instructors. Both instructors followed the same training protocol, educational materials, demonstration sequence, and practical exercises to ensure consistency across all sessions.

### 2.4. Assessment of Chest Compression Quality

Following completion of the training, participants individually performed uninterrupted chest compressions for two minutes on an AmbuMan^®^ Basic CPR manikin (Ambu A/S, Ballerup, Denmark) according to current ERC and AHA Basic Life Support guidelines. Participants were unable to view the manikin feedback monitor during the assessment. Chest compression performance was evaluated using the real-time visual feedback system integrated into the manikin. According to the manufacturer’s performance criteria based on ERC and AHA recommendations, three chest compression quality parameters were assessed: adequate compression rate (100–120 compressions/min), adequate compression depth (5–6 cm), and complete chest recoil [12]. The simulator’s green indicator was recorded as adequate performance and the red indicator as inadequate performance for each predefined parameter. Because the AmbuMan^®^ Basic provides categorical rather than continuous feedback, only categorical outcomes were available for statistical analysis. The simulator underwent routine institutional maintenance and functionality checks, and all participants were assessed using the same simulator under identical testing conditions.

### 2.5. Outcome Measures

The primary outcome was the association between body mass index (BMI) and chest compression quality, assessed using three predefined parameters: adequate compression rate, adequate compression depth, and complete chest recoil. Secondary outcomes included the associations of age, sex, nursing experience, previous BLS training, and employment in an intensive care unit with each of these chest compression quality parameters. These associations were evaluated using multivariable logistic regression analyses.

### 2.6. Statistical Analysis

An a priori sample size calculation was performed using G*Power version 3.1.9.4. Based on previous evidence demonstrating small-to-moderate correlations between BMI and chest compression performance among nurses [8], a conservative expected correlation coefficient of r = 0.20 was selected for the sample size calculation. This correlation-based effect size was used as a general estimate of the expected association between BMI and chest compression performance during the study planning stage, before the final multivariable logistic regression models were specified. With a two-sided significance level of α = 0.05 and a statistical power of 80% (1 − β = 0.80), the minimum required sample size was calculated as 194 participants. To account for potential missing data or incomplete assessments, additional participants were recruited, resulting in a final sample of 284 nurses.

Statistical analyses were performed using IBM SPSS Statistics, version 24.0 (IBM Corp., Armonk, NY, USA). Data distribution was assessed using the Kolmogorov–Smirnov and Shapiro–Wilk tests. Normally distributed continuous variables are presented as mean ± standard deviation (SD), whereas non-normally distributed variables are expressed as median (interquartile range, IQR). Categorical variables are presented as frequencies and percentages.

Continuous variables were compared among BMI groups using one-way analysis of variance (ANOVA) or the Kruskal–Wallis test, as appropriate. When the Kruskal–Wallis test indicated statistical significance, pairwise comparisons were performed using the Dunn–Bonferroni post hoc test. Categorical variables were analyzed using the chi-square test or Fisher’s exact test. For categorical variables showing overall statistical significance, pairwise comparisons were adjusted using the Bonferroni correction.

Multivariable logistic regression analysis was performed to evaluate the independent association between BMI, entered as a continuous variable, and each chest compression quality parameter (adequate vs. inadequate). Age, sex, nursing experience, previous BLS training, and working in an intensive care unit were included as covariates in the regression models. Results are presented as adjusted odds ratios (aORs) with 95% confidence intervals (95% CIs). Multicollinearity among the independent variables was assessed using variance inflation factors (VIFs). A VIF value < 3 was considered to indicate the absence of problematic multicollinearity. All statistical tests were two-sided, and a *p*-value < 0.05 was considered statistically significant.

## 3. Results

A total of 284 nurses were included in the study. There were no missing data for any of the study variables. The participants had a median age of 26.0 years (IQR, 25.0–29.8), a mean height of 164.9 ± 6.6 cm, a median body weight of 60.0 kg (IQR, 54.3–68.0), and a median body mass index (BMI) of 22.5 kg/m^2^ (IQR, 20.6–24.9). The median duration of professional nursing experience was 24.5 months (IQR, 16.0–60.0).

Of the participants, 82.7% were female. Most held a bachelor’s degree (84.2%), while 77.5% were working in general wards, 19.0% in intensive care units, and 3.5% in other clinical departments. Regarding previous Basic Life Support (BLS) training, 26.4% of the participants reported that they had never received BLS training, whereas 32.0% stated that their most recent training had been completed more than one year before the study.

The demographic characteristics, professional background, and training-related data of the participants are summarized in Table 1.

Comparisons of chest compression quality parameters across the BMI groups demonstrated significant differences in the proportions of participants achieving adequate compression rate, adequate compression depth, and complete chest recoil (Table 2).

The proportion of participants achieving an adequate compression rate was 15.0% (3/20), 78.4% (152/194), and 91.4% (64/70) in the BMI < 18.5 kg/m^2^, BMI 18.5–24.9 kg/m^2^, and BMI ≥ 25 kg/m^2^ groups, respectively (χ^2^ = 52.016, *p* < 0.001). Post hoc comparisons showed significantly lower rates in the BMI < 18.5 kg/m^2^ group than in both higher BMI groups, with no significant difference between the BMI 18.5–24.9 kg/m^2^ and BMI ≥ 25 kg/m^2^ groups (Table 2).

Adequate compression depth was achieved by 45.0% (9/20), 84.0% (163/194), and 88.6% (62/70) of participants in the three BMI groups, respectively (χ^2^ = 21.474, *p* < 0.001). Similarly, the BMI < 18.5 kg/m^2^ group had significantly lower rates than both higher BMI groups, whereas the latter two groups did not differ significantly (Table 2).

Complete chest recoil was achieved by 65.0% (13/20), 88.1% (171/194), and 92.9% (65/70) of participants, respectively (χ^2^ = 11.296, *p* = 0.004). Post hoc comparisons again showed significantly lower rates in the BMI <18.5 kg/m^2^ group than in both higher BMI groups, with no significant difference between the two higher BMI groups (Table 2).

Multivariable logistic regression analysis was performed to evaluate the independent association between BMI and chest compression quality (Table 3). After adjustment for age, sex, nursing experience, previous BLS training, and working in an intensive care unit, BMI remained independently associated with adequate compression rate (adjusted OR = 1.32, 95% CI: 1.18–1.49; *p* < 0.001) and adequate compression depth (adjusted OR = 1.29, 95% CI: 1.14–1.47; *p* < 0.001). In contrast, BMI was not independently associated with complete chest recoil (adjusted OR = 1.12, 95% CI: 0.99–1.26; *p* = 0.072). No evidence of problematic multicollinearity was identified, with VIF values ranging from 1.05 to 2.04.

## 4. Discussion

In this prospective simulation study, a significant association was observed between body mass index (BMI) and chest compression quality. Participants with a BMI below 18.5 kg/m^2^ were less likely to achieve the recommended compression rate and compression depth than those in the higher BMI categories. In contrast, no significant differences in these parameters were found between participants with a BMI of 18.5–24.9 kg/m^2^ and those with a BMI of ≥25 kg/m^2^. Although differences in complete chest recoil were identified among the BMI groups, BMI was not independently associated with recoil after adjustment for potential confounding variables.

Our findings indicate that individuals with a lower BMI were less successful in achieving adequate compression depth and compression rate. These results are generally consistent with those reported in previous simulation studies. Küçükceran et al. demonstrated significantly lower mean compression depth and a lower proportion of adequate compression depth among participants with a BMI below 21 kg/m^2^ [5]. Likewise, Roh and Lim reported that nursing students with a BMI below 18.5 kg/m^2^ achieved significantly lower compression depth than participants with higher BMI values [13]. Similarly, Eyupoglu et al. found that nurses who performed optimal CPR had a higher median BMI than those providing suboptimal CPR and that participants who achieved adequate compression depth had greater body weight [6]. The authors suggested that increased body weight may facilitate the generation of sufficient force required to achieve the recommended compression depth. Consistent with these observations, Więch et al. reported positive associations between height, body weight, BMI, and both compression depth and compression rate in a large simulation study involving 505 nurses [8]. Our findings support these previous observations, as participants in the lowest BMI group demonstrated significantly lower rates of adequate compression depth and compression rate. However, the absence of significant differences between the BMI 18.5–24.9 kg/m^2^ and BMI ≥ 25 kg/m^2^ groups is consistent with a possible threshold pattern, whereby the association between BMI and chest compression performance may be more apparent at lower BMI values. This interpretation should be considered hypothesis-generating and warrants further investigation.

The mechanisms underlying the poorer chest compression performance observed in participants with a lower BMI remain uncertain. Nevertheless, individuals with lower BMI may have a reduced capacity to generate and sustain the force required to deliver effective chest compressions. Previous studies have shown that greater body weight, height, and BMI are associated with increased compression depth, probably because these characteristics allow rescuers to transfer greater vertical force to the patient’s sternum during compressions. Gianotto-Oliveira et al. and Reddy et al. reported deeper chest compressions among overweight rescuers, whereas López-González et al. found that underweight participants were less likely to achieve the recommended compression depth than those with normal or higher BMIs [14,15,16]. Similarly, Więch et al. demonstrated positive correlations between body weight, height, BMI, and compression depth in nurses [8]. The same study further showed that fat-free mass, skeletal muscle mass, and total body water were positively associated with compression depth, suggesting that body composition, in addition to BMI, may contribute to CPR performance. Although body composition was not evaluated in our study, the lower rates of adequate compression depth and compression rate observed in the low-BMI group may reflect a reduced ability to generate and maintain sufficient compression force throughout the resuscitation effort. Nevertheless, BMI should be interpreted as an indirect anthropometric marker rather than the true physiological determinant of CPR performance. Individuals with similar BMI values may differ substantially in lean body mass, skeletal muscle mass, upper limb strength, and overall physical fitness, all of which may directly influence the ability to generate effective chest compressions. Therefore, the observed association between BMI and chest compression quality may partly reflect these underlying physiological characteristics rather than the effect of BMI itself. Given the observational design of the study, these findings should be interpreted as associations and do not establish a causal relationship between BMI and chest compression performance. Future studies incorporating direct measurements of body composition and muscle strength are needed to clarify the mechanisms linking physical characteristics with CPR quality.

Więch et al. also reported an inverse association between higher BMI and complete chest recoil [8]. In our study, although complete chest recoil differed significantly among BMI categories, post hoc comparisons showed lower recoil rates in the BMI < 18.5 kg/m^2^ group than in both higher BMI groups, with no significant difference between the BMI 18.5–24.9 kg/m^2^ and BMI ≥ 25 kg/m^2^ groups. However, BMI was not independently associated with complete chest recoil after adjustment for potential confounding variables. These findings suggest that the relationship between BMI and complete chest recoil may differ from that observed for compression rate and depth and should therefore be interpreted cautiously.

One of the strengths of the present study is its prospective design and relatively large sample of 284 nurses, which supports the robustness of the findings within the study setting. In addition, all participants received the same standardized Basic Life Support training before performance assessment, ensuring that chest compression quality was evaluated under comparable conditions. Furthermore, chest compression quality was assessed using three predefined quality parameters—compression rate, compression depth, and complete chest recoil—rather than a single composite outcome, allowing a more comprehensive evaluation of the relationship between BMI and CPR performance.

Several limitations should be acknowledged. First, this was a single-center simulation study; therefore, the findings may not be directly generalizable to real-life cardiac arrest situations. Second, although participants were recruited consecutively from the hospital’s annual in-service BLS training program, the voluntary nature of participation may have introduced selection bias. Third, only BMI was evaluated as an anthropometric marker, whereas lean body mass, skeletal muscle mass, upper extremity strength, fat-free mass, and other body composition variables, which may more directly influence chest compression quality, were not measured. Fourth, CPR performance was assessed immediately after training, and long-term retention of chest compression skills was not evaluated. Fifth, rescuer fatigue during the two-minute chest compression assessment was not formally evaluated and therefore could not be considered in the analysis. Finally, the a priori sample size calculation was based on a correlation-based effect size rather than assumptions specific to multivariable logistic regression with binary outcomes. Therefore, the study may not have been optimally powered for all adjusted binary-outcome models, and this should be considered when interpreting the multivariable findings.

The present findings may be relevant to simulation-based CPR training programs. Participants with lower BMI demonstrated lower rates of achieving the recommended chest compression rate and depth under standardized training conditions. Routine simulation-based performance assessment may help identify healthcare professionals who experience difficulty achieving guideline-recommended chest compression quality, allowing individualized feedback and additional supervised practice when needed. However, given the single-center simulation design, the applicability of these findings to broader healthcare populations and real-life resuscitation settings requires further investigation.

## 5. Conclusions

Participants with a BMI below 18.5 kg/m^2^ demonstrated lower rates of adequate compression depth and compression rate than those with higher BMI values. However, no significant differences in these parameters were observed between the BMI 18.5–24.9 kg/m^2^ and BMI ≥ 25 kg/m^2^ groups. This finding is suggestive of a possible threshold pattern in the association between BMI and chest compression performance and should be interpreted cautiously. In addition, BMI was not independently associated with complete chest recoil. Taken together, these findings suggest that particular attention may be warranted for healthcare professionals with lower BMI during Basic Life Support training and chest compression performance assessment.

## Figures and Tables

**Table 1:** Baseline demographic, professional, and training characteristics of the study participants (*n* = 284).

Characteristic	Value
Age, years	26.0 (25.0–29.75)
Height, cm	164.89 ± 6.59
Weight, kg	60.0 (54.25–68.0)
Body mass index, kg/m^2^	22.49 (20.57–24.89)
Nursing experience, months	24.5 (16.0–60.0)
Sex, *n* (%)	
Female	235 (82.7)
Male	49 (17.3)
Education level, *n* (%)	
Bachelor’s degree	239 (84.2)
Postgraduate degree	26 (9.2)
High school	19 (6.7)
Working unit, *n* (%)	
General wards *	220 (77.5)
Intensive care units †	54 (19.0)
Other clinical units ‡	10 (3.5)
Previous BLS training, *n* (%)	
No previous training	75 (26.4)
<3 months	24 (8.5)
3–6 months	50 (17.6)
6–12 months	44 (15.5)
>1 year	91 (32.0)

Data are presented as median [interquartile range], mean ± standard deviation, or number (%), as appropriate. * General wards include adult and pediatric inpatient wards. † Intensive care units include adult and pediatric intensive care units. ‡ Other clinical units include the operating room, emergency department, and palliative care unit.

**Table 2:** Comparison of chest compression quality parameters according to body mass index categories.

Chest Compression Quality Parameter	BMI < 18.5 kg/m^2^ (*n* = 20)	BMI 18.5–24.9 kg/m^2^ (*n* = 194)	BMI ≥ 25 kg/m^2^ (*n* = 70)	χ^2^	*p*
Correct compression rate	3 (15.0) ^a^	152 (78.4) ^b^	64 (91.4) ^b^	52.016	<0.001
Correct compression depth	9 (45.0) ^a^	163 (84.0) ^b^	62 (88.6) ^b^	21.474	<0.001
Complete chest recoil	13 (65.0) ^a^	171 (88.1) ^b^	65 (92.9) ^b^	11.296	0.004

Data are presented as n (%), with percentages calculated within each BMI category. Overall comparisons were performed using Pearson’s chi-square test. Post hoc pairwise comparisons were performed using separate Pearson’s chi-square tests with Bonferroni correction for multiple comparisons; therefore, *p* < 0.0167 was considered statistically significant. Values within the same row that do not share a superscript letter differ significantly. BMI, body mass index.

**Table 3:** Multivariable logistic regression analysis of factors associated with chest compression quality parameters.

Independent Variable	Adequate Compression Rate, aOR (95% CI);	*p*	Adequate Compression Depth, aOR (95% CI);	*p*	Complete Chest Recoil, aOR (95% CI);	*p*
BMI, per 1 kg/m^2^ increase	1.32 (1.18–1.49)	<0.001	1.29 (1.14–1.47)	<0.001	1.12 (0.99–1.26)	0.072
Age, per year	0.91 (0.81–1.02)	0.101	1.00 (0.88–1.13)	0.981	0.93 (0.82–1.06)	0.275
Sex, male vs. female	0.60 (0.26–1.40)	0.237	0.51 (0.21–1.24)	0.139	2.94 (0.64–13.38)	0.164
Nursing experience, per months	1.01 (1.00–1.02)	0.106	1.00 (0.99–1.01)	0.623	1.00 (0.99–1.02)	0.397
Previous BLS training (yes vs. no)	0.74 (0.36–1.52)	0.411	0.77 (0.35–1.69)	0.515	0.75 (0.31–1.86)	0.541
Working in an intensive care unit (yes vs. no)	2.10 (0.89–4.96)	0.090	2.28 (0.87–5.98)	0.093	2.49 (0.72–8.60)	0.150

Adjusted models included age, sex, nursing experience, previous BLS training, and working in an intensive care unit. ORs for BMI represent the change in odds per 1 kg/m^2^ increase in BMI.

## Data Availability

The data presented in this study are available from the corresponding author upon reasonable request. The data are not publicly available due to ethical restrictions and participant privacy considerations.
